# Feasibility and Efficacy of Mobile Three-Dimensional c-Arm Systems for Single-Stage Localization and Resection of Small Pulmonary Nodules: A Pilot Clinical Trial

**DOI:** 10.1093/icvts/ivaf313

**Published:** 2025-12-22

**Authors:** Hsin-Yueh Fang, Chuan Cheng, Pin-Li Chou, Yin-Kai Chao

**Affiliations:** Division of Thoracic Surgery, Chang Gung Memorial Hospital, College of Medicine, Chang Gung University, Taoyuan 333, Taiwan; Division of Thoracic Surgery, Chang Gung Memorial Hospital, College of Medicine, Chang Gung University, Taoyuan 333, Taiwan; Division of Thoracic Surgery, New Taipei Municipal Tu-Cheng Hospital, New Taipei City 236, Taiwan; Division of Thoracic Surgery, Chang Gung Memorial Hospital, College of Medicine, Chang Gung University, Taoyuan 333, Taiwan; Division of Thoracic Surgery, Chang Gung Memorial Hospital, College of Medicine, Chang Gung University, Taoyuan 333, Taiwan

**Keywords:** small pulmonary nodules, localization, resection, 3D C-arm systems

## Abstract

**Objectives:**

Hybrid operating rooms (HORs) incorporating robotic C-arm systems facilitate concurrent localization and resection of small pulmonary nodules, albeit with significant cost barriers. Contemporary mobile 3 D C-arm systems may provide superior soft tissue visualization with enhanced economic viability and accessibility. This prospective single-arm clinical pilot trial was designed to assess the technical feasibility, clinical efficacy, and procedural safety of employing mobile 3 D C-arm technology for single-stage localization and excision of small pulmonary nodules.

**Methods:**

Patients presenting with small and/or deep-seated lung tumors necessitating preoperative localization were eligible for inclusion. Two distinct mobile 3 D C-arm systems (Cios Spin and Ziehm Vision RFD 3 D) were employed. The primary end-points included the rate of successful tumour localization and the time required to complete the localization procedure. Secondary end-points encompassed perioperative complications and radiation exposure.

**Results:**

The study cohort included 41 patients with lung tumors measuring a median diameter of 7.30 mm (interquartile range [IQR]: 5.00-9.20 mm) and a median depth of 10.00 mm (IQR: 2.00-18.00 mm). Successful localization was achieved in 40 patients, yielding a success rate of 97.6%. In one case, inadequate lesion visualization using the mobile 3 D C-arm necessitated localization in a hybrid operating room. The mean localization time was 27.17 ± 10.38 min, and the median radiation exposure was 531.04 μGy m^2^ (IQR: [297.12-870.98] μGy m^2^). All patients were successfully discharged, with a median postoperative hospital stay of 3 days (IQR: 3-3 days).

**Conclusions:**

Our results support the technical feasibility, clinical efficacy, and procedural safety of mobile 3 D C-arm systems for single-stage localization and resection of small pulmonary nodules.

**Clinical trial registration number:**

*ClinicalTrials.gov* identifier: NCT04974632.

## Graphical abstract

**Figure:**
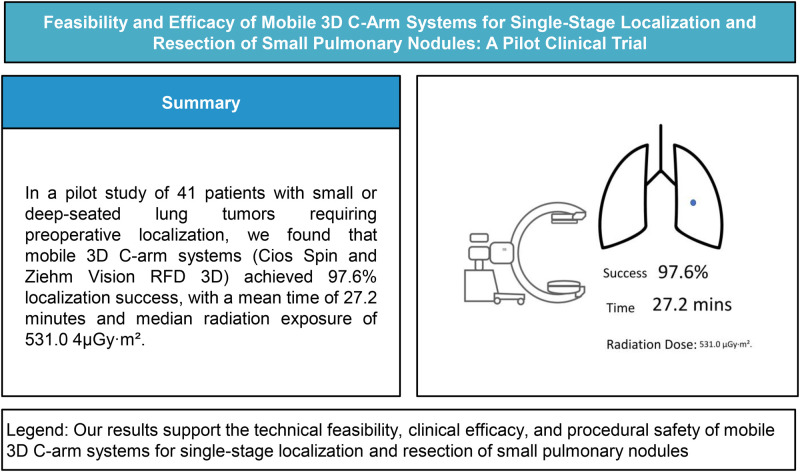

## INTRODUCTION

Thoracoscopic wedge resection of small or deeply situated pulmonary nodules poses a significant challenge for thoracic surgeons due to difficulties in accurate localization. Failure to precisely identify these lesions can result in unsuccessful thoracoscopic procedures, often necessitating conversion to open thoracotomy.^1^ Consequently, precise tumour localization is essential for achieving a successful surgical outcome.^2^

Traditionally, ensuring accurate nodule localization and removal involves a two-stage protocol, wherein marking procedures are performed in a radiology suite before patient transfer to the operating room. However, this sequential process extends the time-at-risk—defined as the interval between needle puncture and surgical intervention—potentially exacerbating patient discomfort and anxiety while increasing vulnerability to procedure-related complications.^3^ In response to these limitations, an emerging single-stage approach that integrates localization and surgery in a hybrid operating room (HOR) has demonstrated significant reductions in time-at-risk without compromising procedural success rates.^3^^,^^4^ Despite these clinical advantages, the substantial capital investment required for HOR facilities presents a significant economic barrier to widespread implementation.^5^^,^^6^

Recent technological advances have yielded mobile 3 D C-arm systems capable of high-contrast soft tissue imaging.^7–9^ While this technology potentially represents a cost-effective alternative that addresses current procedural limitations in pulmonary nodule management, empirical evidence validating its clinical utility remains insufficient. To address this knowledge gap, we conducted a pilot clinical trial evaluating the technical feasibility, clinical efficacy, and procedural safety of mobile 3 D C-arm-guided single-stage localization and excision of small pulmonary nodules.

## METHODS

### Study design

This study was conducted as a prospective, single-arm clinical pilot trial, following the ethical principles delineated in the Declaration of Helsinki. All collection and storage of data from research participants for multiple and indefinite use was consistent with requirements outlined in the WMA Declaration of Taipei. The research protocol received approval from the Institutional Review Board and local regulatory authorities at Chang Gung Memorial Hospital-Linkou, Taiwan (reference number: CGMH-IRB 202100442A3) on June 29, 2021. All participants provided written informed consent for the collection, analysis, and publication of their data. Furthermore, the study was registered on *ClinicalTrials.gov* (identifier: NCT04974632).

### Inclusion and exclusion criteria

Inclusion criteria comprised patients with solid pulmonary nodules that were either small (diameter < 10 mm) and/or deeply situated (tumour-to-visceral pleura distance > 15 mm), as well as those exhibiting subsolid patterns (irrespective of size or depth). Patients with a body mass index (BMI) exceeding 30 kg/m^2^ were excluded from the study.

### Mobile 3 D C-arm workflow for small pulmonary nodule localization

**Figure 1.**
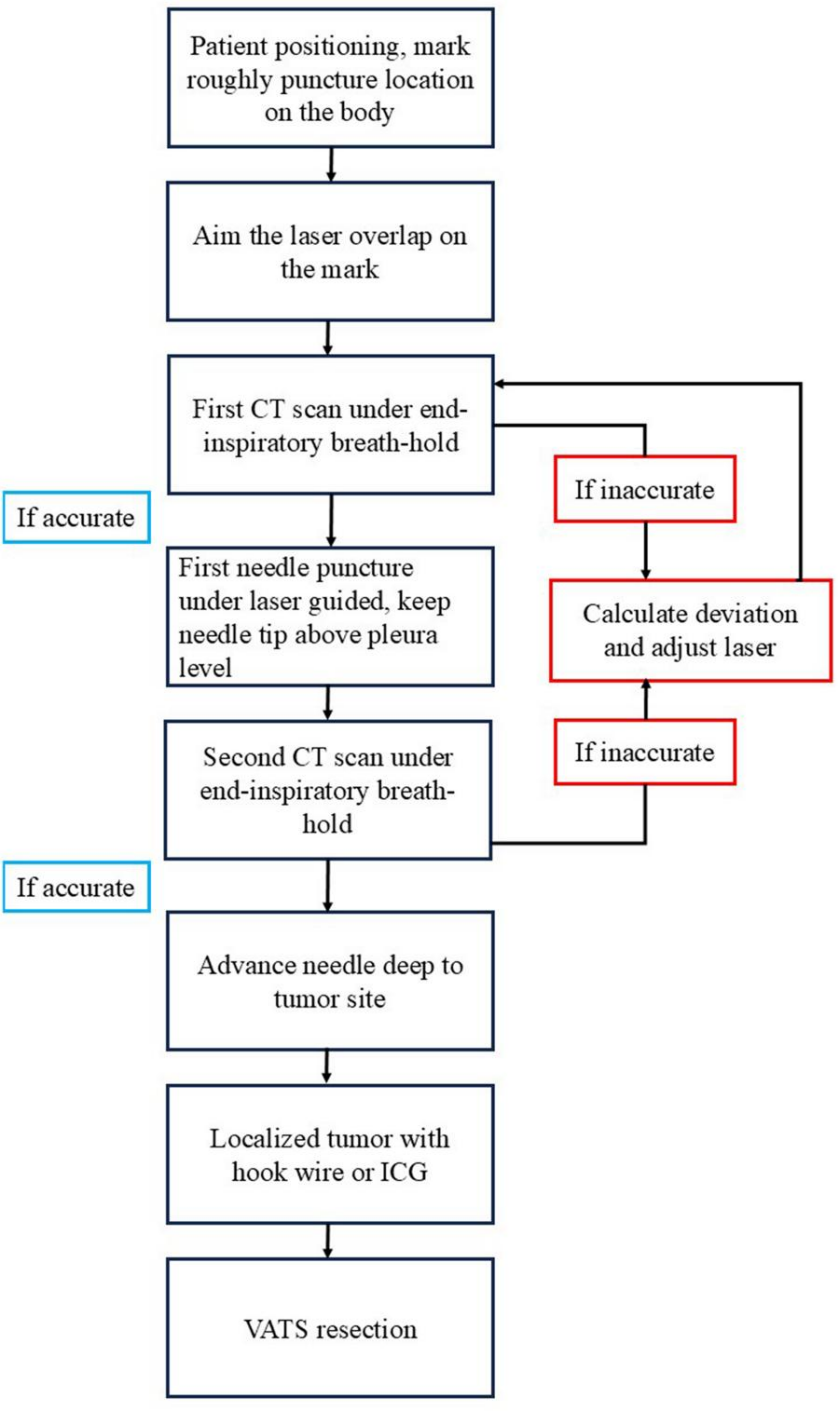
Schematic Workflow of the Nodule Localization Procedure

**Video 1** provides a summary of Mobile 3 D C-arm guided localization. Thoracic surgeons reviewed preoperative computed tomography (CT) scans to establish optimal patient positioning and puncture trajectory. Following general anaesthesia induction and double-lumen endotracheal tube placement, patients were positioned in supine, prone, or lateral decubitus orientation. An initial rotary scan was performed under end-inspiratory breath-hold conditions. In accordance with the study protocol, either the Cios Spin (Siemens Healthineers, Forchheim, Germany) or the Ziehm Vision RFD 3 D (Ziehm Imaging, Nuremberg, Germany) system was employed. As part of the clinical trial, we collaborated with both manufacturers to obtain access to their mobile 3 D C-arm systems. Due to logistical constraints and the loan schedules of the devices, the Cios Spin system was used exclusively during the first half of the study period, followed by the Ziehm Vision RFD 3 D system during the latter half. The puncture path was designed to minimize distance while avoiding transfissural routes and major vascular structures. Under cone-beam computed tomography (CBCT) guidance, an 18-gauge marker needle was incrementally advanced until it contacted the target lesion. Tumour localization was subsequently achieved through the application of either 0.3 ml of indocyanine green dye for superficially situated lesions or hookwire placement for more deeply positioned neoplasms. **Figure 1** provides a comprehensive illustration of the procedural workflow. As shown in **Video 1**, the standard procedure included 3 rotations: the initial rotation demonstrated the target and allow us to design puncture route; the second rotation to make sure the accuracy of puncture route; the third rotation to confirm the final needle location. As our experience grew and technology matured, the third rotation was not necessary if the second CT scan revealed accurate route.

All procedures were performed by a single team of thoracic surgeons who were experienced in image-guided localization in HOR. During the CT scan, the C-arm operator, along with all surgical and anaesthesia team members, will remain behind a lead shield to minimize radiation exposure.

### Data collection and variable definition

Each study participant was assigned a unique identification code. Access to the identification registry was strictly limited to study coordinators. All data collection and storage procedures were conducted in accordance with Good Clinical Practice guidelines. Physical data were stored in locked cabinets at designated data coordinating centers, with access restricted to the principal investigator, research nurses, and authorized physicians.

### Study end-points

The primary end-points comprised the rate of successful targeting during localization and the localization procedure time. Successful targeting was defined as the localization needle being placed within 2 cm of the lesion. The technical success rate of targeting was defined as the proportion of successful localization procedures relative to the total number of localization attempts. Secondary end-points included procedure-related complication rates, defined as the incidence of adverse events (pneumothorax, haemothorax, or parenchymal Haemorrhage) directly attributable to needle insertion. Pneumothorax was further categorized according to the 2010 British Thoracic Society guidelines as large (> 2 cm distance between lung margin and chest wall) or small (≤ 2 cm). Radiation exposure was quantified using dose area product (DAP), expressed in μGy m^2^. Additional variables of interest included demographic and tumour characteristics, operative findings, and complications. Lesion size was defined as the maximum diameter measured on the axial view, while lesion depth was calculated as the minimum distance from the lesion center to the pleural surface on preoperative CT scans. Complication severity was assessed using the Clavien-Dindo classification system, with grade IIIa or higher designated as severe.

### Statistical analysis

Continuous variables are presented as medians and interquartile ranges (IQRs). Categorical variables are reported as frequencies and percentages. For an exploratory comparison between the two mobile 3 D C-arm systems, we employed the Wilcoxon rank-sum test for continuous variables and Fisher’s exact test for categorical data. Analyses were performed using SPSS, version 20.0, with all tests two-sided at a 5% level of significance.

## RESULTS

### Cohort demographics and tumour characteristics

**Figure 2.**
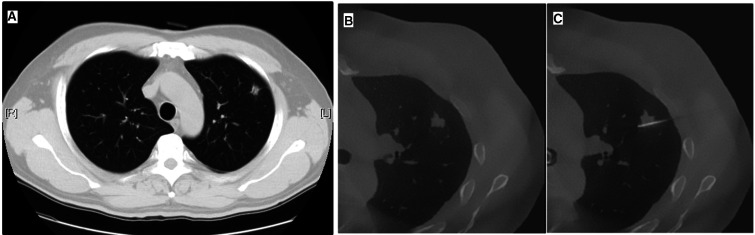
Representative Images of a Right Upper Lobe Pulmonary Nodule. Panel A Displays a Preoperative Traditional CT Image. Panel B Shows a Cone Beam Computed Tomography Image Obtained Prior to Localization. Panel C Illustrates the Image Captured during the Localization Procedure

**Table 1** depicts the cohort demographics and tumour characteristics. Between July 2021 and August 2022, 41 patients (20 men and 21 women) were enrolled, with a mean age of 55.22 ± 11.26 years and a median BMI of 23.15 kg/m^2^ (IQR: 21.16-25.79 kg/m^2^). Preoperative CT imaging demonstrated a median tumour size of 7.30 mm (IQR: 5.00-9.20 mm) and a median pleural depth of 10.00 mm (IQR: 2.00-18.00 mm), yielding a median depth-to-size ratio of 1.22 (IQR: 0.27-2.25). Nodule morphology was classified as solid in 23 patients (56.1%) and subsolid in 18 participants (43.9%). Localization procedures employed either the Cios Spin apparatus (*n* = 20) or the Ziehm Vision RFD 3 D system (*n* = 21). **Figure 2** presents representative images acquired using multidetector computed tomography and mobile 3 D C-arm CBCT, accompanied by the corresponding localization imaging findings.

**Table 1.** General Characteristics of the Study Patients (*n* = 41)

Age, years; mean (SD)	55.22 (11.26)
Sex, *n*	
Men/women	20/21
Body mass index, Kg/m^2^; median (IQR)	23.15 (21.16-25.79)
ASA physical status classification, *n*	
I/II/III	1/11/29
CT findings, *n*	
Solid nodule/Subsolid nodule	23/18
Lesion size on CT imaging, mm; median (IQR)	7.30 (5.00-9.20)
Lesion location, *n*	
RUL/RML/RLL/LUL/LLL	11/5/9/9/7
Distance to the pleural space, mm; median (IQR)	10.00 (2.00-18.00)
Depth-to-size ratio; median (IQR)	1.22 (0.27-2.25)

Abbreviations: SD = standard deviation; IQR = interquartile range; ASA = American Society of Anesthesiologists; CT = computed tomography; RUL = right upper lobe; RML = right middle lobe; RLL = right lower lobe; LUL = left upper lobe; LLL = left lower lobe.

#### Primary end-points

**Table 2** summarizes the details of the localization procedures. Of the total cohort, 12 patients (29.3%) underwent localization in the supine position, 5 (12.2%) in the prone position, and 24 (58.5%) in the lateral decubitus position. Localization was successfully accomplished in 40 of the 41 study participants, yielding an overall technical success rate of 97.6%. The mean duration of the localization procedure was 27.17 ± 10.38 min.

**Table 2.** Procedural Characteristics of Pulmonary Nodule Localization Using Mobile 3 D C-Arm Systems in the Study Patients (*n* = 41)

Localization time, min; mean (SD)	27.17 (10.38)
Patient position during localization, *n*	
Supine/prone	12/5
Lateral decubitus	24
Localization marker, *n*	
Hookwire/indocyanine green dye	16/25
Mobile 3 D C-arm system, *n*	
Cios spin/Zeihm	20/21
Number of CT scans; median (IQR)	3 (2-4)
Radiation dose, μGy·m^2^; median (IQR)	531.04 (297.12-870.98)
Operative procedure, *n*	
Wedge resection	37
Wedge resection -> Lobectomy	2
Segmentectomy	2
Clavien-Dindo Classification 0/1/2/3 A/3B/4	38/2/0/0/1/0
Postoperative LOS, days; median (IQR)	3 (3-3)
Distance between nodule and surgical margin, mm; median (IQR)	10.37 (6.25-13.75)
Final pathological diagnosis, *n*	
Primary lung cancer/lung metastasis/benign l	20/5/16

Abbreviations: SD = standard deviation; IQR = interquartile range; CT = computed tomography; LOS = length of stay.

**Figure 3.**
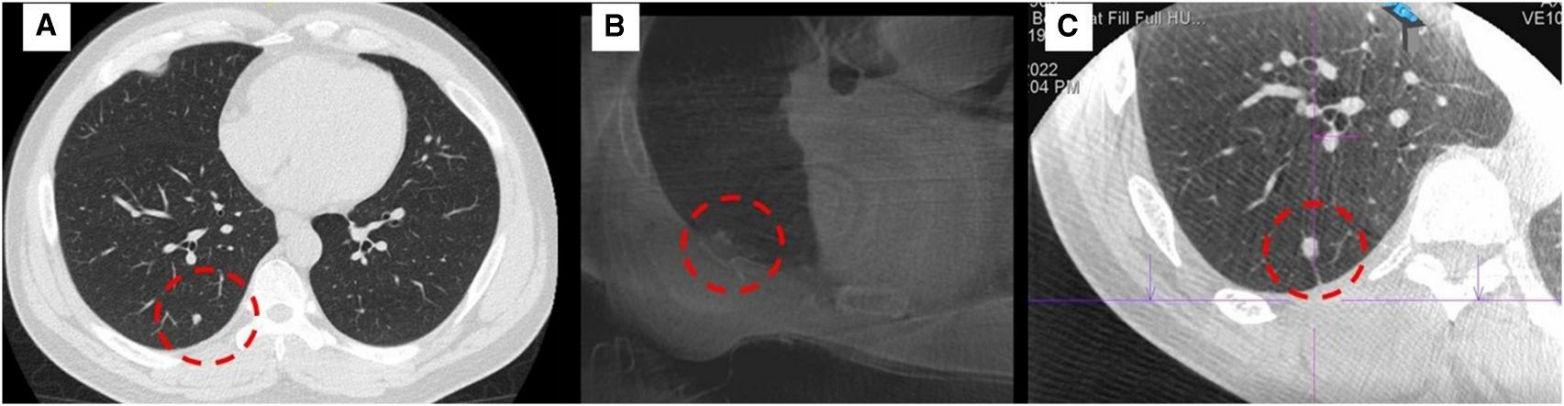
Imaging Comparison in the Failed Prone Localization Case. The red-dotted circle indicates the location of lesions. (A) Preoperative CT image demonstrating the pulmonary lesion. (B) Intraoperative 3D C-arm image acquired with the patient in prone position on a foam-and-gel surgical pad. The lesion could not be clearly identified due to compromised image quality, likely caused by the pad attenuating X-ray penetration. (C) Cone-beam CT (CBCT) image obtained in the hybrid operating room (HOR) after repositioning, which successfully delineated the target lesion and allowed for accurate localization

**Figure 3A** shows the original CT image of the only failed case, in which the right lower lobe lesion could not be clearly visualized using the 3 D C-arm system (**Figure 3B**). As a result, the procedure was transferred to CBCT-guided localization in the HOR (**Figure 3C**). We believe the main factor contributing to the poor image quality was the surgical support pad placed beneath the patient’s torso. This pad, made of foam and gel to prevent pressure injuries, likely interfered with X-ray penetration and led to image degradation.

#### Secondary end-points

The median number of CT scans required for nodule localization was 3 (IQR: 2-4), with a median DAP of 531.04 μGy m^2^ (IQR: 297.12-870.98 μGy m^2^). No procedure-related complications, such as pneumothorax or haemothorax, were observed. All 41 patients who underwent localization (40 with mobile 3 D C-arm and 1 with CBCT in the HOR) successfully received VATS resection without conversion to thoracotomy. The initial surgical plan included wedge resection in 39 cases and segmentectomy in 2 cases, due to concerns about insufficient deep margins. Among the 39 patients who initially underwent wedge resection, 2 ultimately converted to lobectomy. One was due to an inadequate deep margin for a right middle lobe mass, and the other was converted based on the surgeon’s intraoperative suspicion of visceral pleural involvement. Patients with intraoperative frozen-section diagnoses indicative of malignancy or premalignant lesions underwent subsequent systematic lymph node dissection. The final pathology result showed 20 primary lung malignancy, 5 metastases, and 16 benign lesions; the malignancy rate was 61% (25/41). Among patients diagnosed with primary lung malignancy, all dissected lymph nodes tested negative for tumour involvement. Therefore, all patients with primary lung malignancy in this cohort were pathologic stage I. No operative-related mortality was documented. Postoperative complications were documented in three patients. Specifically, two experienced grade I events according to the Clavien-Dindo classification, whereas one patient developed a grade IIIB complication. This more severe case presented as chylothorax, which proved refractory to conservative management and ultimately necessitated surgical reintervention for thoracic duct ligation. The median postoperative hospital stay was 3 days (IQR: 3-3 days). All patients completed a full three-year postoperative follow-up. Among those diagnosed with malignancy, no cases of local recurrence were observed during the follow-up period.

## DISCUSSION

To our knowledge, this study represents the first clinical trial investigating the feasibility, safety, and efficacy of mobile 3 D C-arm systems for single-stage localization and resection of small pulmonary nodules. Our findings demonstrate that this technology effectively visualizes small pulmonary lesions, achieving a 97% localization success rate with acceptable procedural duration (median: 27 min). The substantially lower acquisition cost of mobile 3 D C-arm systems (approximately $400,000) compared with HOR setups (approximately $2.6 million) suggesting that they may serve as a practical alternative for single-stage procedures.^6^ Furthermore, the compact footprint of these systems, comparable to conventional C-arm equipment, enables transportation between standard operating rooms, providing an efficient solution for the growing population of patients with early-stage lung cancer requiring localization procedures.

While it is true that the mobile 3 D C-arm system in our study was primarily used to visualize and deliver a localization wire or ICG dye to the target lesion rather than for continuous live screening, we believe this one stage procedure still provides significant clinical and logistical value. Unlike conventional localization performed in the radiology suite followed by patient transfer to the operating room, the use of a mobile 3 D C-arm allows localization and surgery to occur in the single stage setting. This approach avoids time delays, reduces the risk of marker migration during transfer, and eliminates the need for additional coordination between departments. Moreover, although the mobile 3 D C-arm system lacks some advanced imaging features of a HOR, it represents a more economical accessible alternative, especially in institutions where HOR infrastructure is unavailable.

Therefore, we believe the mobile 3 D C-arm offers a favourable balance between functionality, workflow efficiency, and cost—particularly for institutions seeking to streamline single-stage procedures without major investment in HOR facilities.

In this pilot trial, two mobile 3 D C-arm systems were employed. While both platforms achieved comparable localization success rates, procedures using the Ziehm system were more technically demanding and required additional CT acquisition sequences, resulting in prolonged procedural times (**Table 3**). This discrepancy is primarily attributed to differences in the learning curve. Our surgical team had prior hands-on experience with the Spin system before trial initiation, allowing for immediate operational efficiency without a significant adaptation period.^10^ In contrast, the Ziehm platform operates on fundamentally different principles—most notably, a non-isocentric rotational design combining orbital and translational motion—which prevented direct transfer of experience from the Spin system. As a result, additional imaging was necessary during the initial learning phase of Ziehm-guided procedures.

**Table 3.** Exploratory Comparison between the Cios Spin and Ziehm Vision RFD 3 D Mobile C-Arm Systems: patient Characteristics, Technical Parameters, and Study Endpoints

	Cios Spin	Ziehm	*P* value
	n = 20	n = 21	
Age, years; mean (SD)	59.50 (10.49)	51.14 (10.66)	.016
BMI, kg/m^2^; mean (SD)	23.09 (3.54)	23.68 (3.22)	.584
Sex, *n*			
Male	11	9	
Female	9	12	
Tumour size, mm; median (IQR)	7.50 (5.00-8.25)	8.30 (7.00-10.00)	.186
Tumour depth, mm; median (IQR)	12.00 (4.25-20.00)	10.00 (2.00-15.00)	.455
Depth-to-size ratio; median (IQR)	1.24 (0.36-3.28)	1.22 (0.21-1.97)	.547
Type of nodule, *n* (%)			.448
Solid	15 (75%)	13 (62%)	
Subsolid	5 (25%)	8 (38%)	
Localization success, *n*	20	20	
Time required for localization, mean (SD)	24.65 (10.27)	29.57 (10.14)	.131
Number of CT spins, median (IQR)	2 (2-3)	4 (3-4.75)	.001
Radiation exposure, μGy·m^2^; median (IQR)	743.60 (592.08-1075.54)	360.64 (194.28-509.43)	.004
Postoperative LOS, days; median (IQR)	3 (3-3)	3 (3-3)	.677

Abbreviations: SD = standard deviation; IQR = interquartile range; BMI = body mass index; CT = computed tomography; LOS = length of stay.

Notably, despite requiring additional scans, the Ziehm system delivered significantly lower radiation exposure, with median DAP measurements of 360.64 μGy m^2^ compared to 743.60 μGy m^2^ for the Spin apparatus (*P* = .004). Several factors may explain this radiation efficiency advantage (**Table S1**). Accordingly, the Ziehm system operates at lower nominal X-ray power output,^11^ while its non-isocentric rotation potentially optimizes exposure parameters more effectively. Additionally, it incorporates advanced dose optimization algorithms, utilizes a smaller detector footprint requiring less overall radiation, and employs efficient reconstruction techniques that collectively minimize radiation exposure while preserving diagnostic image quality.^12^^,^^13^ These combined features establish the Ziehm system as a more dose-efficient platform for mobile CBCT imaging.^14^

The results of this pilot trial warrant careful interpretation within the context of several limitations. First, our findings were generated by a thoracic surgical team with substantial prior experience in HOR procedures,^4^^,^^15^^,^^16^ potentially limiting generalizability to centers with less specialized expertise. Independent validation across diverse institutions is essential to establish external validity. Second, although our approach demonstrates considerable promise, several technical parameters require further optimization to maximize procedural efficiency and precision. Third, the exclusion of patients with a BMI > 30 kg/m^2^ represents an important limitation of our study. This criterion was prespecified because the mobile 3 D C-arm system has relatively limited X-ray output, and increased soft tissue thickness in patients with a larger body habitus may attenuate the X-ray beam and diminish the visibility of pulmonary nodules. Additionally, the relatively small field of view of the mobile 3 D C-arm may make it difficult to simultaneously include both the skin entry point and the target lesion within the same imaging frame in patients with a larger thoracic cavity, thereby increasing the risk of localization inaccuracy.^17^ Therefore, the applicability of this 3 D C-arm–based localization approach in obese patients remains uncertain and should be validated in future studies. Fourth, although the acquisition cost of mobile 3 D C-arm systems is significantly lower than that of HORs, a true cost-effectiveness analysis was beyond the scope of this study. Such an analysis would require formal health-economic modelling, incorporating long-term clinical outcomes (eg, survival, complications), utility-based measures such as quality-adjusted life years, and sensitivity analyses across diverse clinical scenarios. Additionally, one might question whether the most cost-effective strategy in selected patients—particularly those with low radiologic suspicion for malignancy—is localization and resection at all. In such cases, alternatives such as upfront resection without marking or even active surveillance may offer superior value. These hypotheses merit further investigation in larger comparative effectiveness studies. Finally, this study included multiple exploratory comparisons between two mobile 3 D C-arm systems. These analyses were not prespecified in the original trial protocol and were conducted post hoc. As no adjustments were made for multiple comparisons, the findings should be considered exploratory and interpreted with caution. The absence of correction for multiplicity increases the risk of type I errors, and some observed differences may not be reproducible in future studies. These results are hypothesis-generating and warrant further validation through prospective, adequately powered comparative trials.

## CONCLUSION

Our results support the technical feasibility, clinical efficacy, and procedural safety of mobile 3 D C-arm systems for single-stage localization and resection of small pulmonary nodules.

## Supplementary Material

ivaf313_Supplementary_Data

## Data Availability

The data underlying this article will be shared on reasonable request to the corresponding author.
