# Supplementary material for: Feasibility and Efficacy of Mobile Three-Dimensional c-Arm Systems for Single-Stage Localization and Resection of Small Pulmonary Nodules: A Pilot Clinical Trial
Source: Interdiscip Cardiovasc Thorac Surg. 2025 Dec 22;41(1):ivaf313. doi: 10.1093/icvts/ivaf313 (PMC12957938; doi:10.1093/icvts/ivaf313)
Supplement: ivaf313_Supplementary_Data [file ivaf313_Supplementary_Data.zip › 20250302SupplementalT1.doc]

Supplementary Table 1 General settings of the Cios Spin and Ziehm Vision RFD 3D mobile C-arm systems

|  | Cios Spin | Ziehm |
| --- | --- | --- |
| Power, kW | 12/25 | 25 |
| Tube voltage (kVp) range | 40‒125 | 40‒120 |
| Focal spot size, mm | 0.3/0.5 | 0.3/0.6 |
| Beam filtration options | 3.0 mm aluminum equivalent inherent filtration (measured at 75 kV) + 0.1 mm copper added filtration + 0.3 aluminum equivalent from the dose area product chamber | 0.2 mm copper + at least 1.0 mm aluminum equivalent |
| Frame rate, frames/sec | Up to 30 | 25; 30 |
| Field-of-view, cm | 30 × 30 | 30 × 30 |
| Gantry speed, degree/sec | 6.7 | 5 |
| Source-to-axis distance, cm | 62 | 65 |
| Source-to-detector distance, cm | 116 | 107 |
| Extent of rotation, degrees | 196 | 165  15 additional degrees covered by means of  two translational phases prior to and  after rotation phase; total angular coverage > 180 degrees |
| Number of projections per scan | 100/200/400 | Approximately 400 |
| Scan speed, sec | 30, 60 | 48 |
